# Self-Reactivity and the Expression of Memory Markers Vary Independently in MRL-Mp+/+ and MRL-Mp-*lpr/lpr* Mice

**DOI:** 10.1155/1992/89109

**Published:** 1992

**Authors:** Lesley Smyth, Michelle Howell, I. Nicholas Crispe

**Affiliations:** 1 ICAPB Zoology Building West Mains Road Edinburgh Scotland; 2 University of Glasgow Medical School Glasgow Scotland; 3 Immunobiology Section Yale Medical School 310 Cedar Street New Haven Connecticut 06510 USA

**Keywords:** MRL, *lpr*, T-cell repertoire, CD4+ cell subsets

## Abstract

MRL-Mp-*lpr/lpr* mice contain phenotypically abnormal populations of T cells, and exhibit an SLE-like autoimmune disease in which autoantibodies are a prominent feature. We analyzed the phenotype and T-cell receptor V*ß* expression pattern in CD4^+^ T cells of this mutant mouse strain to detect abnormalities that could explain the autoimmunity. The CD4^+^  T cells contain two distinct abnormal populations. One of these expresses B220 and HSA, and in these and other respects closely resembles the accumulating CD4^–^CD8^–^ population. The other expresses a high level of CD44 (Pgp-1), and a high level of the 16A epitope of CD45, and so resembles post-activation T cells. Both of these cell types are exclusive to MRL-Mp-*lpr/lpr*. We also identified V *ß*5- and V *ß*11-positive CD4+ T cells, in both MRL-Mp-*lpr/lpr* and MRL-Mp-+/+ mice. We conclude that autoimmune T cells can be detected in these mice, but that they are not the cause of the accumulation of abnormal CD4^+^ and CD4^–^CD8^–^cells.


*Developmental Immunology*, 1992, Vol. 2, pp. 309–318
Reprints available directly from the publisher
Photocopying permitted by license only

© 1992 Harwood Academic Publishers GmbH
Printed in the United Kingdom

### Self-Reactivity and the Expression of Memory Markers Vary Independently in MRL-Mp+/+ and MRL-Mp-*lpr*/*lpr* Mice

LESLEY SMYTH,† MICHELLE HOWELL,‡ and I. NICHOLAS CRISPE§*

†*ICAPB, Zoology Building, West Mains Road, Edinburgh, Scotland*
‡*University of Glasgow Medical School, Glasgow, Scotland*
§*Immunobiology Section, Yale Medical School, 310 Cedar Street, New Haven, Connecticut 06510*

MRL-Mp-*lpr*/*lpr* mice contain phenotypically abnormal populations of T cells, and exhibit an SLE-like autoimmune disease in which autoantibodies are a prominent feature. We analyzed the phenotype and T-cell receptor V$\beta$ expression pattern in CD4$^+$ T cells of this mutant mouse strain to detect abnormalities that could explain the autoimmunity. The CD4$^+$ T cells contain two distinct abnormal populations. One of these expresses B220 and HSA, and in these and other respects closely resembles the accumulating CD4$^-$CD8$^-$ population. The other expresses a high level of CD44 (Pgp-1), and a high level of the 16A epitope of CD45, and so resembles post-activation T cells. Both of these cell types are exclusive to MRL-Mp-*lpr*/*lpr*. We also identified V$\beta$5- and V$\beta$11-positive CD4+ T cells, in both MRL-Mp-*lpr*/*lpr* and MRL-Mp-+/+ mice. We conclude that autoimmune T cells can be detected in these mice, but that they are not the cause of the accumulation of abnormal CD4$^+$ and CD4$^-$CD8$^-$ cells.

KEYWORDS: MRL, *lpr*, T-cell repertoire, CD4+ cell subsets.

### INTRODUCTION

The mouse strain MRL-Mp-*lpr*/*lpr* is a mutant strain that exhibits an abnormality of T-cell differentiation, in parallel with a severe SLE-like autoimmune disease (Theofilopoulos and Dixon, 1985). In these animals, there accumulates a bizarre population of CD4$^-$, CD8$^-$ T cells with $\alpha\beta$ antigen receptors, which causes massive enlargement of the peripheral lymphoid organs (Davidson et al., 1986). Cells of identical phenotype have been identified in the thymus and peripheral lymphoid tissue of normal mice, where they form a very minor subpopulation (Fowlkes et al., 1987; Guidos et al., 1989). It has been proposed that the defect in MRL-Mp-*lpr*/*lpr* mice results in the accumulation of an increased number of cells in what is normally a very small T-cell population. Alongside these CD4$^-$, CD8$^-$ cells, MRL-Mp-*lpr*/*lpr* mice contain a number of CD4$^+$ and CD8$^+$ T cells, at least some of which are able to function normally (Simon et al., 1984).

The abnormal function of B cells in MRL-Mp-*lpr*/*lpr* mice results in the production of autoantibodies, which react with a number of self-components including autologous DNA (Dixon, 1987). To test the idea that autoantibody production was due to T-cell help by self-reactive CD4$^+$ T cells, we examined these cells by flow cytometry to look for phenotypic markers of self-reactivity. We studied a number of cell surface markers that are expressed on normal T cells only after their activation in the periphery; and we used antibodies against a set of T-cell receptor V$\beta$ gene products to identify cells with the potential to respond to autologous MHC class II I-E molecules (Gao et al., 1988; Woodland et al., 1990).

In MRL-Mp-*lpr*/*lpr* mice, we confirmed the existence of a population of phenotypically abnormal CD4$^+$ T cells, and characterized these cells in terms of the markers CD45/B220, CD3, CD44 (Pgp-1), and Ly-6C. No such cells were found in the parent strain MRL-Mp-+/+. We

*Corresponding author.

studied the CD45 isoform distribution on $CD4^+$ T cells, and again found patterns restricted to the MRL-Mp-*lpr/lpr* strain. In contrast to studies in mouse strains where the *lpr* gene has been back-crossed onto other genetic backgrounds (Kotzin et al., 1988), we found expression of T-cell receptors including the I-E reactive variable regions $V\beta5$ and $V\beta11$ in some, but not all MRL-Mp-*lpr/lpr* mice. Unlike the abnormalities of CD44 and CD45 expression, this phenomenon was also seen in the parent strain MRL-Mp-+/+.

We conclude that the expression in MRL-Mp-*lpr/lpr* mice of markers of prior exposure to antigen, such as CD44 (Budd et al., 1987) and the CD45 isoform pattern (Bottomly et al., 1989), is not related to the leakage of self-reactive TCR into the periphery, because this occurs also in MRL-Mp-+/+ mice, which have a normal CD44 and CD45 marker phenotype.

### RESULTS

### The B220 Epitope of CD45 Marks Abnormal $CD4^+$ Cells

Lymph node of MRL-Mp-+/+ and MRL-Mp-*lpr/lpr* mice were stained with anti-CD4-PE and anti-B220 followed by FITC goat anti-rat Ig. In 16-week-old MRL-Mp-*lpr/lpr* mice, the lymph nodes are substantially larger than normal, and most of the T cells are $B220^+$, $CD4^-$ (Fig. 1). In CD4 versus CD8 staining, these cells are also $CD8^-$ (data not shown). The mice also contain a population of $B220^+$, $CD4^+$ T cells, as has been previously described (Asano et al., 1988; Kariyone et al., 1988), in addition to a "normal" $B220^-$, $CD4^+$ subset. The MRL-Mp-+/+ parent strain has normal-sized lymph nodes, and contains neither $B220^+$ population of T cells. The $B220^+$, $CD4^+$ cells are not unique to the MRL-Mp-*lpr/lpr* strain, and are also found in B6-*lpr/lpr* mice (L. Smyth, unpublished data).

To determine the relationship between the phenotypically abnormal $CD4^+$ T cells and the course of the disease, lymph node cells of mice at different ages were stained for CD4 versus B220. The total percentage of $CD4^+$ T cells fell steadily from young (6–8 weeks) to older animals (20 weeks), as would be expected. Within the $CD4^+$ T cells, the $B220^+$ subset was rare in young mice, and increased dramatically in parallel with the development of lymphadenopathy between 12 and 16 weeks of age (Fig. 2). However, as the mice approached 20 weeks of age, the proportion of these cells fell again, although the size of the lymph nodes continued to increase. These kinetics are strikingly similar to those of the proliferation of $CD4^-$, $CD8^-$ lymphocytes in the MRL-Mp-*lpr/lpr* liver (Ohteki et al., 1990; Seki et al., 1991). This might suggest that the $B220^+$ subset of $CD4^+$ T cells is an intermediate along an abnormal differentiation pathway that originates in the liver. Lineage-tracing experiments to test this are in progress.

### Other Markers on $B220^+$ $CD4^+$ T cells

Lymph-node cells from older (16–20 weeks) MRL-Mp-*lpr/lpr* mice were stained for three-color FACS analysis with a primary antibody, FITC anti-rat Ig, rat Ig as blocker, and the B220-biotin and CD4-PE followed by Streptavidin-Cascade blue. In three-color FACStar Plus analysis, a gate was set on all $CD4^+$ cells, and B220 was displayed versus other markers (Fig. 3). Both $B220^+$ and $B220^-$ subsets of $CD4^+$ cells expressed comparable levels of CD3, but the two cell subsets were very different in other markers. All $B220^+$ cells, but only a variable proportion of the $B220^-$ cells, expressed high levels of the memory T-cell marker CD44. The $B220^+$ cells were also heterogenous in their expression of Ly-6C, and contained both Ly-6C-high and Ly-6C-low cells. Some of them also expressed the immature thymocyte marker HSA (J11d).

The finding of CD44 on a large population of peripheral $CD4^+$ T cells would be compatible with the idea that these cells had already encountered antigen. In an autoimmune strain of mouse such as MRL-Mp-*lpr/lpr*, this could suggest that these were self-reactive cells either involved in the autoimmune process or rendered anergic post-thymically. To detect other features of prior activation, we examined the pattern of CD45 isoform expression; and to look for potentially autoimmune cells, we attempted to detect the expression of TCR $V\beta$ gene products which would be expected to be deleted from the T-cell repertoire in MRL-Mp-*lpr/lpr* mice because of the expression of an I-E molecule.

A two-color FACS analysis was performed on lymph node T cells of 10–12-week-old C57BL/6, MRL-Mp-+/+ and MRL-Mp-*lpr/lpr* mice. The

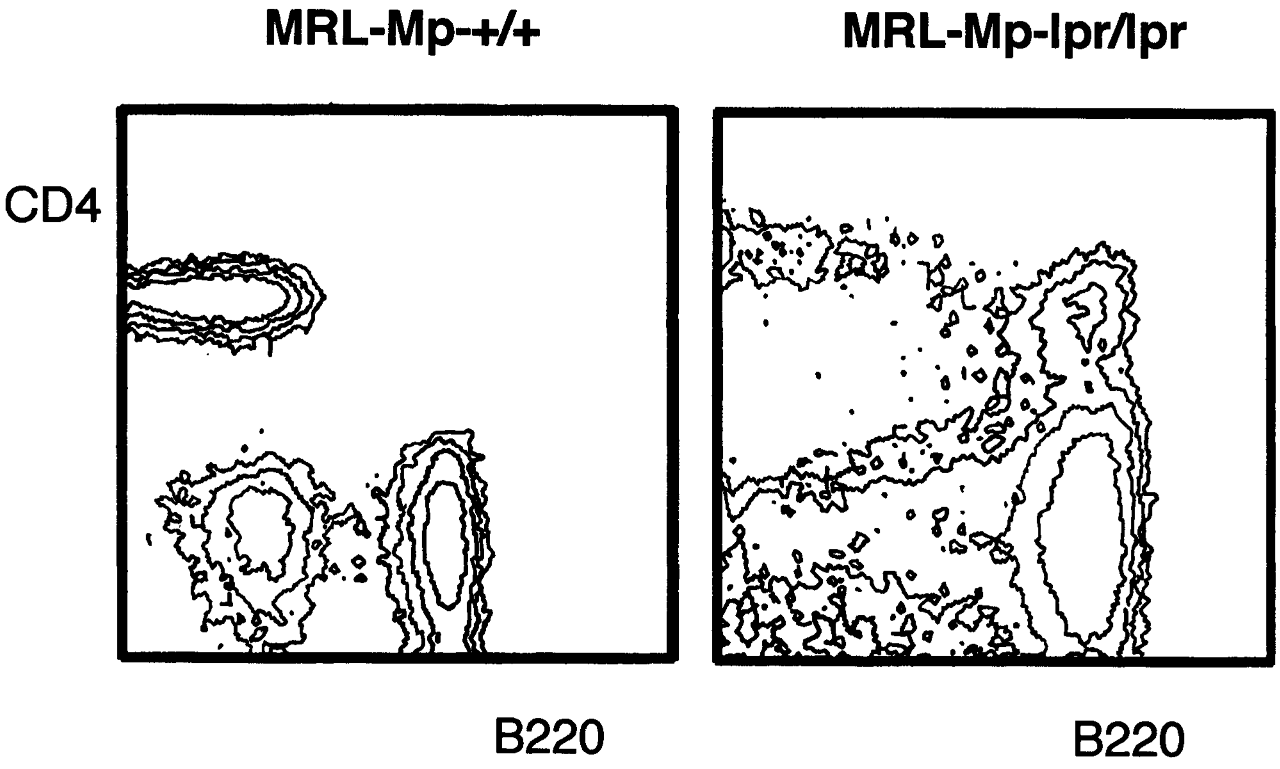

FIGURE 1. Lymph-node cells of MRL-Mp-+/+ and MRL-Mp-*lpr/lpr* mice, stained with CD4 versus B220. The MRL-Mp-*lpr/lpr* cells are mainly B220$^+$, CD4$^-$ (and CD8$^-$), but CD4$^+$ T cells also exist. A subset of these are B220$^+$. Such CD4$^+$, B220$^+$ T cells were also seen in B6-MRL-*lpr/lpr* congenic mice, but not in C57BL/6 or other normal strains.

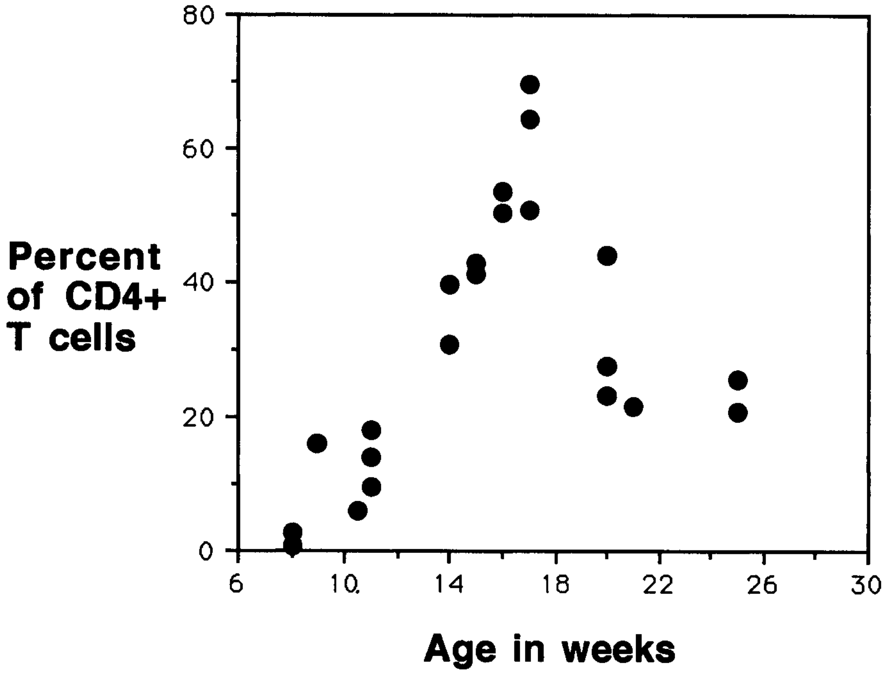

FIGURE 2. The percentage of B220$^+$ cells among CD4$^+$ T cells of MRL-Mp-*lpr/lpr* mice at different ages. The sharp "spike" at around 15 weeks of age coincides with the proliferative potential of liver CD4$^-$, CD8$^-$ cells described by others (Ohteki et al., 1990).

expression pattern on $CD4^+$ T cells of a panel of markers associated with T-cell maturation and homing is summarized in Fig. 4. In this analysis, approximately 13% (range 9–17%) of MRL-Mp-*lpr/lpr* $CD4^+$ T cells were $B220^+$. Such cells are undetectable in the lymph nodes of normal mice. The C57BL6 and MRL-Mp-+/+ cells were very similar for all of the markers studied. The MRL-Mp-*lpr/lpr* $CD4^+$ T cells were different; a lower percentage expressed the lymphocyte homing receptor MEL-14, and many more expressed a high level of CD44 (Pgp-1).

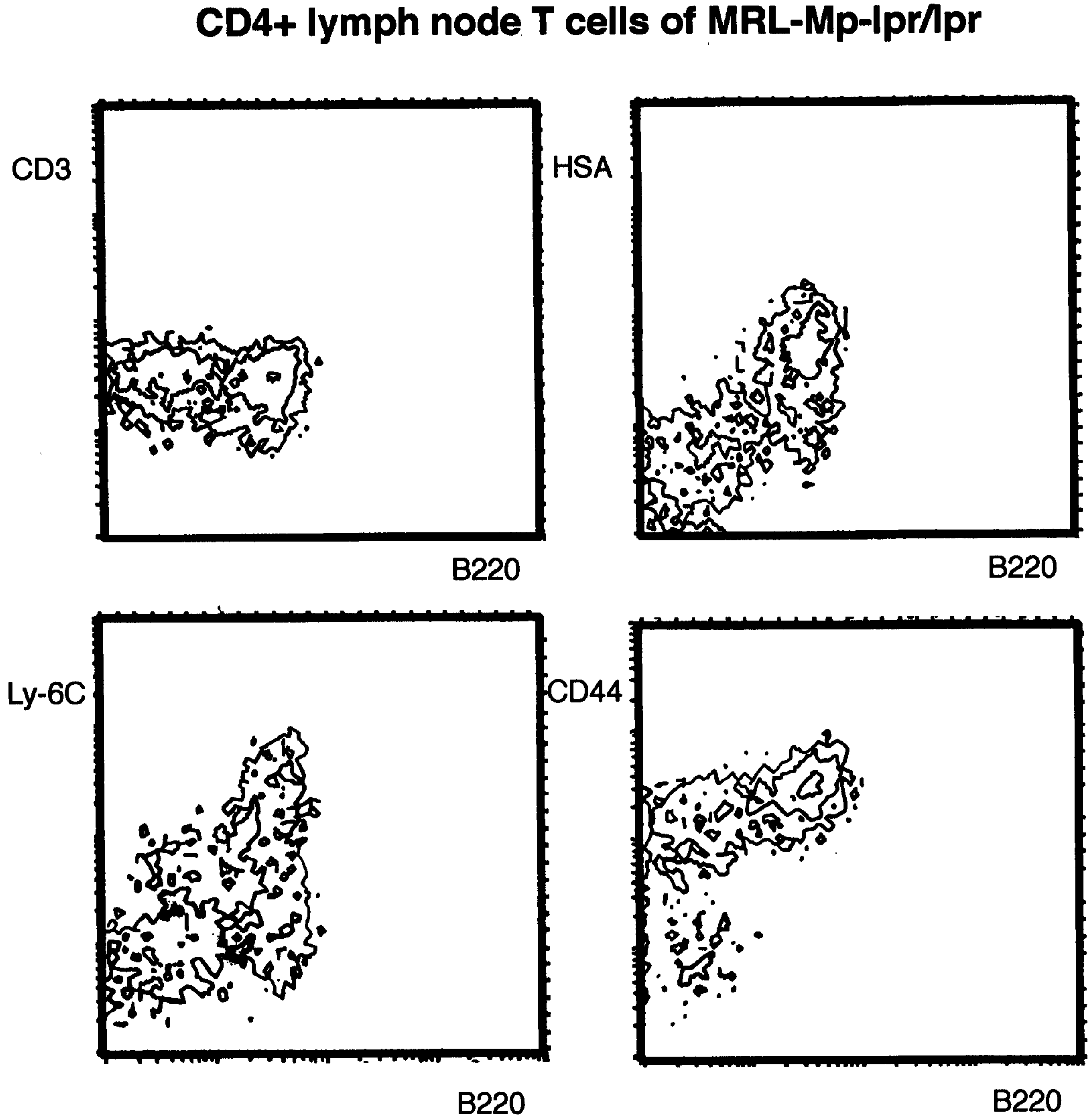

FIGURE 3. Three-color FACS analysis of $CD4^+$ T cells in MRL-Mp-*lpr/lpr*. Cells were stained for CD4 versus B220 and a third marker. The displays are gated on $CD4^+$ cells only, and show B220 versus the other markers. The $B220^+$, $CD4^+$ cells were distinctive in their expression of HSA and high levels of Ly-6C.

The pattern of CD45 exon expression was studied with antibodies that define sets of CD45 isoforms. The monoclonal antibody 16A detects an epitope on higher-molecular-weight isoforms of CD45, in which the exons B and C are spliced in. In the normal mouse strains, high expression of 16A occured on a minority of $CD4^+$ cells; a majority express an intermediate level. In MRL-Mp-*lpr*/*lpr* almost half of the $CD4^+$ T cells were 16A-high, and the 16A-intermediate population was correspondingly smaller. The antibodies 3J and 1F are markers of the B and C exon of CD45 (K. Bottomly, personal communication). Both are negative on normal $CD4^+$ T cells, but these markers were expressed in MRL-Mp-*lpr*/*lpr* at exactly the same frequency as the B220 glycosylation-dependent epitope.

In summary, the lymph nodes of MRL-Mp-*lpr*/*lpr* mice contain three populations of $CD4^+$ T cells: a highly abnormal population with a marker phenotype ($B220^+$, CD44-high, $J11d^+$, $1F^+$, $3J^+$) similar to the accumulating $CD4^-$, $CD8^-$ cells; a population that resembles normal resting T cells in other mouse strains; and an enlarged population that lacks the markers B220, J11d, 1F, and 3J, but expressed high levels of the markers CD44 and the 16A epitope of CD45.

### The $CD4^+$ Cell TCR V$\beta$ Repertoire

Groups of adult (6–20' week) mice were stained for two-color FACS analysis of the expression of various TCR V$\beta$ regions on $CD4^+$ T cells. Data presented are for the strains C57BL6 ($n$=8), AKR/J ($n$=10), MRL-Mp-+/+ ($n$=16), and MRL-Mp-*lpr*/*lpr* ($n$=20), and were obtained by staining animals individually, and pooling data from several separate staining experiments. The C57BL/6 strain expresses neither I-E nor the endogenous retrovirally encoded superantigen Mls-$1^a$, and therefore did not delete V$\beta$5, V$\beta$6, or V$\beta$11 from the T-cell repertoire. The AKR/J strain is H-$2^k$ (I-E+), like MRL-Mp-+/+ and MRL-Mp-*lpr*/*lpr*, and would be expected to delete both V$\beta$5 and V$\beta$11 T cells. This strain also expresses Mls-$1^a$, and therefore would be expected to delete V$\beta$6 also. Our staining experiments confirmed these expectations.

Both the MRL-Mp-*lpr*/*lpr* and the parent strain MRL-Mp-+/+ are Mls-$1^b$ and do not delete V$\beta$6. However, they are both I-E+, which in most cases leads to the deletion of T cells that express V$\beta$5 and V$\beta$11. Surprisingly, expression of both of these "forbidden" TCR V$\beta$ regions was observed in both parent MRL-Mp-+/+ and mutant MRL-Mp-*lpr*/*lpr* mouse strains. Still more surprisingly, the mice were highly variable in their level of expression. Some animals expressed V$\beta$5 in 3–4% of their $CD4^+$ cells, and some appeared to have fully deleted V$\beta$5. Similarly, up to 6% of V$\beta$11+ $CD4^+$ cells were seen in some mice, and others were V$\beta$11-negative. This phenomenon was unrelated to age, and was seen in both young (6–10 week) and older (16–20 week) animals of both affected strains. Because both MRL-Mp-+/+ and MRL-Mp-*lpr*/*lpr* are inbred strains (Murphy and Roths, 1977), genetic in homogeneity is an unlikely explanation for this phenomenon and we are left with two plausible hypotheses: an environmental effect acting on only some of the mice, or a low-frequency stochastic process that sometimes but not always affects V$\beta$5- and/or V$\beta$11-expressing self-reactive T cells.

### DISCUSSION

In the MRL-Mp-*lpr*/*lpr* mouse, self-reactivity and T cells with abnormal phenotypes exist alongside one another. These phenomena could be related if the abnormal T cells result from the diversion of self-reactive T cells into a differentiation pathway that normally exists only as a safety net for the few autoreactive T cells that escape clonal deletion in the thymus. With these ideas in mind, several groups have studied the clonal deletion of various TCR V$\beta$ gene products in mice in which the *lpr* gene has been back-crossed onto genetic backgrounds in which clonal deletion of V$\beta$8.1 or V$\beta$17a can be observed (Kotzin et al., 1988). The consensus result of these studies was that the *lpr* gene does not cause a defect in clonal deletion, although other authors have reached different conclusions (Mountz et al., 1990; Matsumoto et al., 1991).

We initially studied clonal deletion by examining the expression of V$\beta$5 and V$\beta$11 in the MRL-Mp-*lpr*/*lpr* strain, and found evidence for the appearance of these I-E reactive TCR V regions in the $CD4^+$ T-cell population. However, in a strain survey, the appearance of these V regions was a property of background genes present in the parent strain, MRL-Mp-+/+. Clonal deletion of T cells expressing V$\beta$5 or V$\beta$11 depends on the

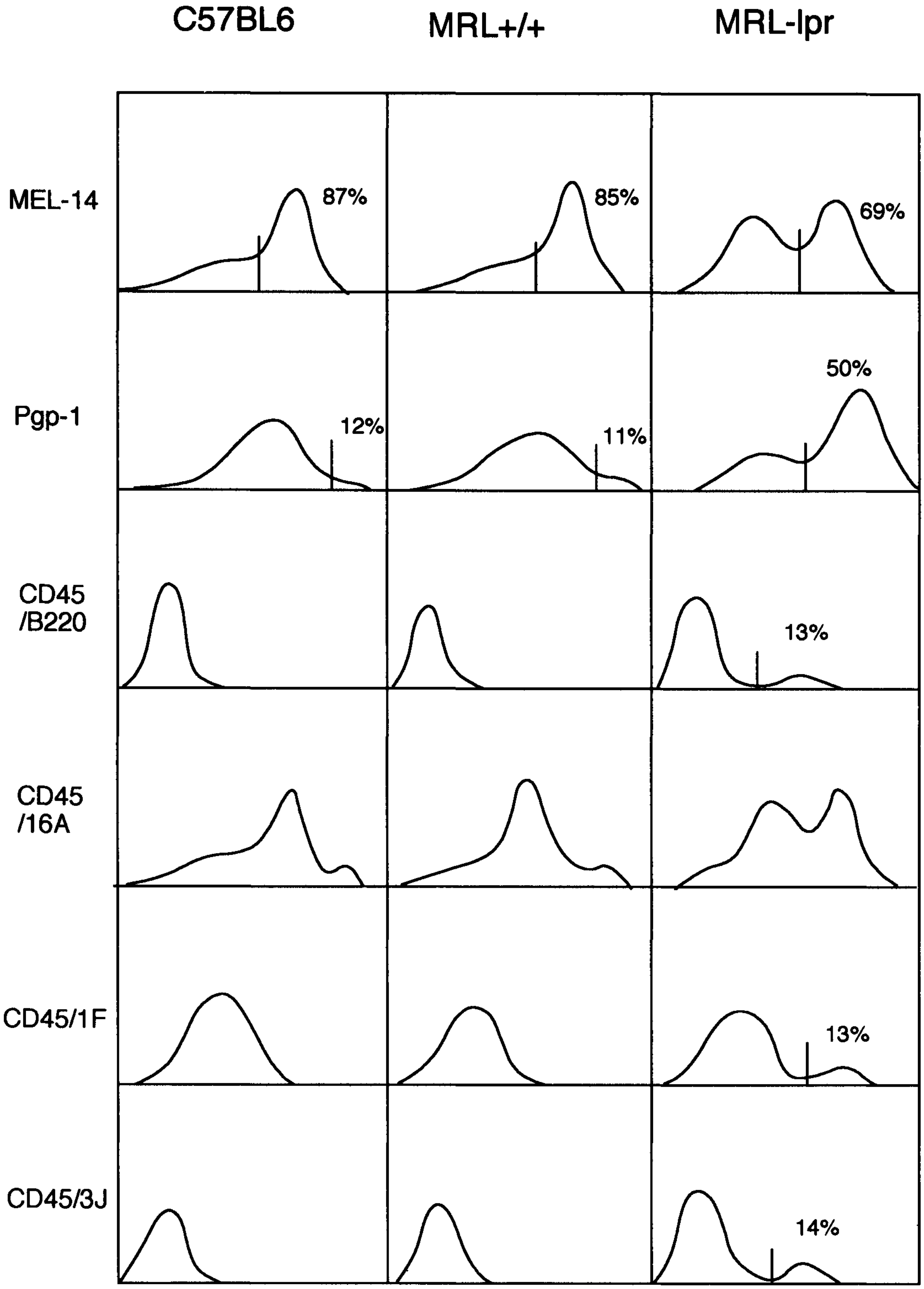

FIGURE 4. Expression of various markers of cell homing and activation on CD4$^+$ lymph-node T cells of a normal mouse strain, versus MRL-Mp-+/+ and MRL-Mp-*lpr*/*lpr*. Cells were stained for two-color analysis, and the histograms show the expression of the antigens gated on CD4$^+$ cells only. Marker bars were set, and percentages calculated, to emphasize the distinctive cell populations observed only in MRL-Mp-*lpr*/*lpr*. For 16A, it is difficult to place such a marker, and the characteristic feature of MRL-Mp-*lpr*/*lpr* cells is the shift of many cells toward a high level of expression.

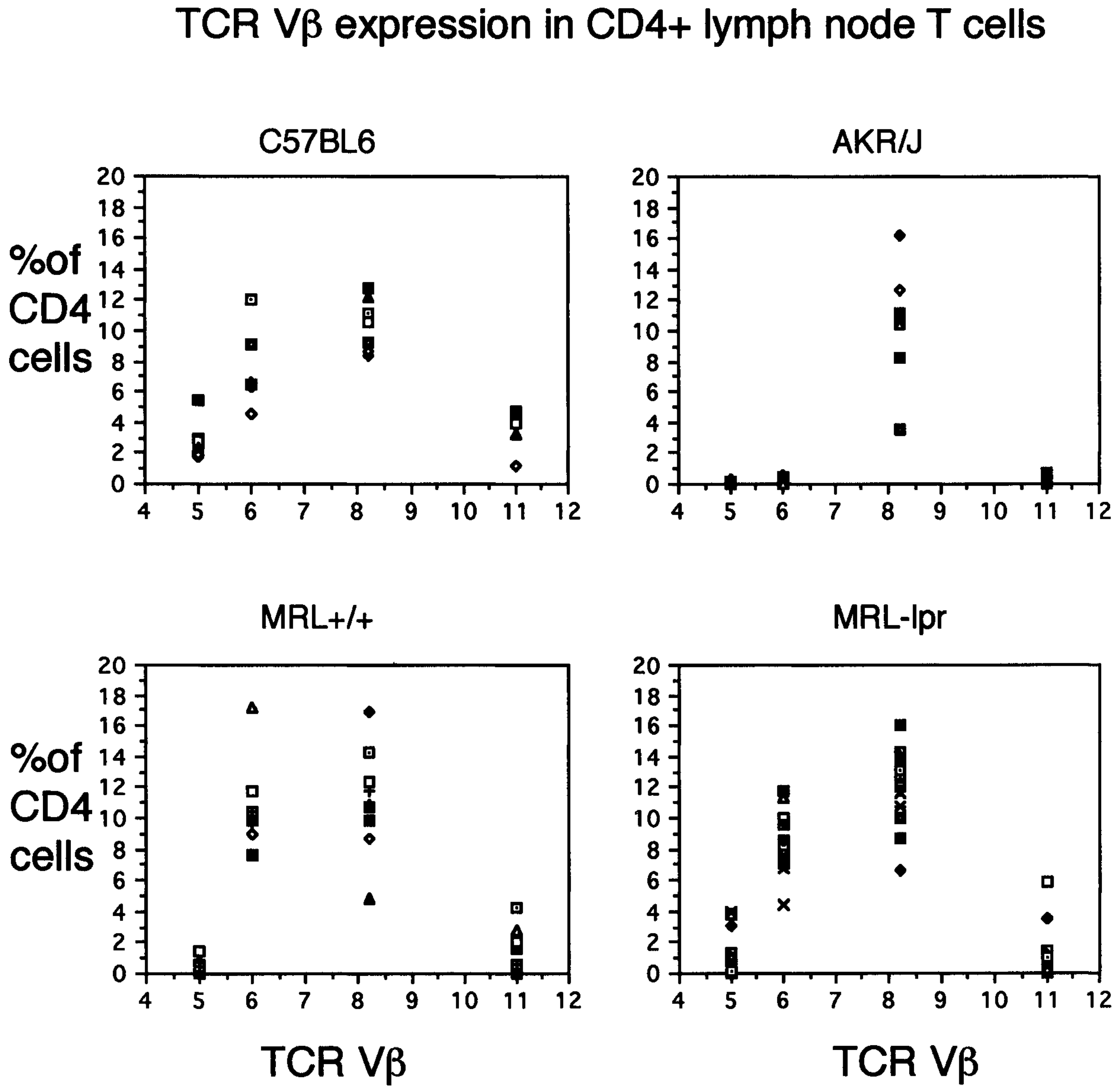

FIGURE 5. Expression of a panel of TCR V$\beta$s on CD4$^+$ cells of individual mice from two normal strains, C57BL6 and AKR/J, and from MRL-Mp-+/+ and MRL-Mp-*lpr*/*lpr*. Lymph-node cells were stained for two-color analysis of CD4 versus various TCR V$\beta$ chains. In AKR/J, the presence of MHC class II molecules plus co-tolerogens including Mls-1$^a$ results in the deletion of V$\beta$5, V$\beta$6, and V$\beta$11. In MRL-Mp-+/+ and MRL-Mp-*lpr*/*lpr*, V$\beta$5 and V$\beta$11 T cells are not deleted despite the presence of I-E.

interaction of developing T cells with both an MHC molecule and a "co-tolerogen" (Bill et al., 1989; Woodland et al., 1990). Many such genetic elements that select a particular set of TCR V$\beta$s have been identified as the product of open reading frames in the LTRs of endogenous retroviruses (Janeway, 1991). Despite the correlation with autoimmune disease, the failure to delete V$\beta$5 and V$\beta$11 in the MRL-Mp-+/+ and MRL-Mp-*lpr*/*lpr* strains could be due to the lack of their appropriate co-tolerogen, rather than a defect in the mechanism of clonal deletion. However, one striking feature of our data is the individual variation between animals of the same strain. All but one of the identified co-tolerogens are endogenous MMTV, and these proviruses are transmitted as autosomal dominant characters. Because both MRL-Mp-+/+ and MRL-Mp-*lpr/lpr* are inbred strains, it is difficult to believe that their co-tolerogen genes could still be segregating. In other cases of TCR Vβ deletion, the lack of co-tolerogen leads to an all-or-none effect in any one mouse strain. However, we cannot exclude the possibility that the individual variation that we observe in Vβ5 and Vβ11 expression is due to a co-tolerogen encoded by an unknown exogenous retrovirus, which is endemic to these mouse strains.

Although the expression of Vβ5 and Vβ11 in the context of H-$2^k$ was a shared property of the MRL-Mp-+/+ and MRL-Mp-*lpr/lpr* mouse strains, the abnormal expression of a panel of B-cell markers on a subset of $CD4^+$ T cells was found exclusively in MRL-Mp-*lpr/lpr* mice. In addition, only MRL-Mp-*lpr/lpr* mice exhibited an expanded population of $CD4^+$ cells with the phenotype of memory cells, characterized by a high level of CD44 expression.

The expression of distinct epitopes of the CD45 molecules defines subsets of thymocytes and peripheral T cells. In the periphery, differences in the pattern of CD45 isoforms defined by the expression of none, one, or several of the variably spliced A, B, and C exons distinguish between $CD4^+$ cells with a Th1-like pattern of cytokine expression and Th2-like cells; and between $CD4^+$ cells which do and do not carry T-cell memory (Bottomly et al., 1989). The display of CD45 isoforms on $CD4^+$ cells of MRL-Mp-*lpr/lpr* mice was studied with three anti-CD45 antibodies: 16A, 3J, and 1F. The antibody 16A recognizes CD45 isoforms which include the B exon, as judged by the recognition of fibroblasts transfected with constructs expressing various known isoforms. This marker is expressed at an intermediate level on primary $CD4^+$ T cells and at a lower level on memory cells (Bottomly et al., 1989). The antibody 3J also recognizes the domain encoded by the B exon, and the antibody 1F requires only the expression of the C exon (T. Novak and K. Bottomly, personal communication). Nevertheless, these last two antibodies do not recognize normal $CD4^+$ T cells. This implies that post-translational modification determines the expression of the 16A, 3J, and 1F epitopes. In the MRL-Mp-*lpr/lpr* mouse, CD44 expression suggests that many $CD4^+$ T cells have been activated, while the large number of cells with very high 16A expression indicates the expansion of a cell population which is very rare in normal mice. The staining with 3J and 1F, together with B220 expression on exactly the same percentage of $CD4^+$ T cells, indicates that not only CD45 exon expression but also post-translational modification is unique in a population of MRL-Mp-*lpr/lpr* T cells.

Our data show that the abnormal differentiation of T cells in mice homozygous for the *lpr* gene cannot be explained simply as a consequence of self-reactive cells in the periphery. Instead, we have evidence for a phenotypically unusual subset of $CD4^+$ T cells, which exhibits the CD44 "memory" phenotype, but the CD45 "naive" phenotype. It is possible that these cells are activated but unable to proceed with normal maturation, and are arrested at an intermediate developmental stage.

It has already been proposed that MRL-Mp-*lpr/lpr* mice contain two parallel pathways of T-cell development: one that predominate in early life and gives rise to phenotypically and functionally normal cells, and one that becomes more prominent as the mouse matures, and generates abnormal cells including the $CD4^-$ $CD8^-$ cells (Budd et al., 1987). Our data support this model of *lpr* pathogenesis. We have also defined a previously unknown major $CD4^+$ T-cell population exclusive to MRL-Mp-*lpr/lpr* mice, which could act as an intermediate in the abnormal pathway. These are the $CD44^+$, 16A-high cells. To make a lineage model that includes these cells, we propose that they differentiate through the $B220^+$, $HSA^+$, Ly-$6C^-$, and the $B220^+$, $HSA^+$, Ly-$6C^+$ subsets, and finally down-regulate the CD4 molecule to become one of the accumulating $CD4^-$, $CD8^-$, $B220^+$, $HSA^+$, and Ly-$6C^+$ cells. The time course of the expression of the $B220^+$, $CD4^+$ phenotype and the steady accumulation of the $B220^+$, $CD4^-$, $CD8^-$ cells suggests that the former may be short-lived intermediates, and the latter long-lived end products of this pathway. A major role for a $CD4^+$ cell population as the origin of the $CD4^-$, $CD8^-$ cells is supported by the striking observation that anti-CD4 monoclonal antibody suppresses the development of both the lymphadenopathy and the autoimmune disease (Santoro et al., 1988; Zhou et al., 1991).

More speculatively, the expansion of the $CD44^+$, 16A-high population of $CD4^+$ cells could explain the acceleration of autoantibody synthesis in MRL-Mp-*lpr*/*lpr* mice. Cells of this phenotype develop *in vitro* after Con-A stimulation, and produce abundant IL-4 (T. Owens, personal communication). If this subset in fact produces IL-4 *in vivo*, and overlaps with the subset of $CD4^+$ T cells that express self-reactive specificities, the mechanism of enhanced autoantibody production in MRL-Mp-*lpr*/*lpr* mice could be the interaction of self-MHC class II I-E reactive $CD4^+$ T cells (including V$\beta$5 and V$\beta$11 cells in some animals) with I-E expressed on autologous B cells. The lineage abnormality associated with the *lpr* mutation would make available an enlarged pool of cells with the $CD44^+$, 16A-high phenotype to participate in such interactions.

A model has been proposed that links the abnormal $CD4^+$ T-cell subsets in mice with the *lpr* mutation into an alternative T-cell maturation pathway. As with the prediction of lineage relationships in the thymus of normal mice, the construction of a hypothetical lineage by juxtaposing cells that show a gradation of phenotypes carries only limited conviction. The differentiation pathway proposed here needs to be tested by direct lineage-tracing experiments.

### MATERIALS AND METHODS

### Animals.

Mice were obtained from the Jackson Laboratories, Bar Habor, ME, or were produced in the Yale Immunobiology Mouse Unit under SPF conditions. Female animals were used unless otherwise stated, at various ages between 6 and 24 weeks.

### Staining Protocol.

Single-cell suspensions were stained according to variations of the following protocol: (a) an unconjugated primary antibody; (b) a fluorochrome-conjugated anti-immunoglobulin, usually FITC-coupled goat anti-mouse Ig or goat anti-rat Ig; (c) a blocking step, using normal serum Ig from either mouse, rat, or hamster to match the antibodies used in subsequent staining steps; (d) directly fluorochrome-coupled or biotinylated reagents; and (e) a Streptavidin reagent. Typically, $10^6$ cells were stained in 50 $\mu$l of staining buffer (PBS containing 1% w/v bovine albumin and 0.02% w/v sodium azide). Cells were stained on ice for 30 min at each stage, except for anti-TCR reagents, which were left for 60 min. Between staining steps, cells were washed with 5.0 ml of staining buffer. In most experiments, cells were fixed in 2% paraformaldehyde and stored at 4°C until analyzed.

### Flow Cytometry.

Two-color analyses were peformed on a FACScan (Becton-Dickinson), using FACScan research software to acquire data. Analysis was on an off-line workstation using Lysys software. Three-color analysis was performed using a dual-laser FACStar Plus (Becton-Dickinson) on cells stained with FITC, PE, and Cascade Blue. Excitation was by two argon ion lasers, one a 3 W using the 488 nm emission line, and the other a 5 W using all lines of UV. Live gating on FSC and SSC was used to exclude dead cells.

### Sources of Reagents.

The hybridoma producing antibody MR-5.1 (anti-V$\beta$5) was made available by Dr. Ed Palmer; RR4-7 (anti-V$\beta$6) and RR3-15 (anti-V$\beta$11) were from Dr. Osami Kanagawa; F23.2 (anti-V$\beta$8.2) was from Dr. Michael Bevan. The antibodies RA3-3A1 (anti-B220) and 145-2C11 (anti-CD3$\varepsilon$) were provided by Dr. B.J. Fowlkes. The HK1.4 (anti-Ly-6C) antibody was provided by Dr. Alfred Bothwell, IM7.8.1 (anti-CD44) was from Dr. H. Robson MacDonald, and J11d (anti-HSA) was from Dr. Jonathan Sprent. The antibodies MEL-14, 16A, 1F, and 3J were obtained from the Yale Hybridoma Facility managed by Dr. Kim Bottomly. FITC-coupled goat anti-mouse Ig and anti-rat Ig were from Sigma; anti-CD4-PE was from Becton-Dickinson: Streptavidin-PE was from Biomeda; and Streptavidin-Cascade blue was from Molecular Probes. All other fluorescent conjugates, and all biotinylated reagents, were prepared in the lab by standard techniques (Katona et al., 1983).

### ACKNOWLEDGMENTS

We wish to thank Drs. M. Bevan, A. Bothwell, K. Bottomly, B.J. Fowlkes, O. Kanagawa, H.R. MacDonald, E. Palmer, and J. Sprent, all of whom made materials available, and Dr. K. Bottomly for constructive criticism of the manuscript. Some of the data compiled in Fig. 2 were obtained by Dennis P.M. Hughes. The work was supported by NIH grant AI 30561-01 to I.N.C, and a Lupus Foundation of America Gina Finzi Summer Studentship to M.H.

*(Received November 27, 1991)*

*(Accepted March 17, 1992)*

### REFERENCES

Asano T, Tomooka S, Serushago B.A., Himeno K., and Nomoto K.A. (1988). A new T cell subset expressing B220 and CD4 in lpr mice: Defects in the response to mitogens and in the production of IL-2. Clin. Exp. Immunol. **74:** 36–40.

Bill J., Kanagawa O., Woodland D., and Palmer E. (1989). The MHC molecule I-E is necessary but not sufficient for the clonal deletion of Vβ11 bearing T cells. J. Exp. Med. **169:** 1405–1409.

Bottomly K., Lugman M., Greenbaum L., Carding S., West J., Pasqualini T., and Murphy D.B. (1989). A monoclonal antibody to murine CD45R distinguishes CD4 T cell populations that produce different cytokines. Eur. J. Immunol. **19:** 617–623.

Budd R.C., Cerottini J.-C., and MacDonald H.R. (1987). Phenotypic identification of memory cytolytic T lymphocytes in a subset of Lyt-2+ cells. J. Immunol. **138:** 1009–1013.

Budd R.C., Schreyer M., Miescher G.C., and MacDonald H.R. (1987). T cell lineages in the thymus of lpr/lpr mice. Evidence for parallel pathways of normal and abnormal T cell development. J. Immunol. **139:** 2200–2210.

Davidson W.F., Dumont F.J., Bedigian H.G., Fowlkes B.J., and Morse H.C. III. (1986). Phenotypic, functional, and molecular genetic comparisons of the abnormal lymphoid cells of C3H-lpr/lpr and C3H-gld/gld mice. J. Immunol. **136:** 4075–4084.

Dixon F.J. (1987). Basic elements of murine systemic lupus erythematosus. J. Rheumatol. **14:** 3–10.

Fowlkes B.J., Kruisbeek A.M., Ton-That H., Weston M.A., Coligan J.E., Schwartz R.H., and Pardoll D.M. (1987). A novel population of T-cell receptor αβ bearing thymocytes which predominantly expresses a single β gene family. Nature **329:** 251–254.

Gao E.-K., Lo D., Cheney D., Kanagawa O. and Sprent J. (1988). Abnormal differentiation of thymocytes in mice treated with Cyclosporin A. Nature **336:** 176–179.

Guidos C.J., Weissman I.L., and Adkins B. (1989). Developmental potential of $CD4^-8^-$ thymocytes. Peripheral progeny include mature $CD4^-8^-$ T cells bearing αβ T cell receptor. J. Immunol. **142:** 3773–3780.

Janeway C.A. (1991). Mls: Makes a little sense. Nature (News and Views) **349:** 459–460.

Kariyone I., Takigushi M., Igarashi S., and Kano K. (1988). Ontogeny and Function of B220+ L3T4+ T cell subset of MRL-Mp-*lpr/lpr* Mice. Cell. Immunol. **115:** 112–120.

Katona I.M., Urban J.F., Scher I., Kanellopoulos-Languevin C., and Finkelman F.D. (1983). Induction of an IgE response in mice by Nippostrongylus brasiliensis: Characterization of lymphoid cells with intracytoplasmic or surface IgE. J. Immunol. **130:** 350–356.

Kotzin B.L., Babcock S.K., and Herron L.R. (1988). Deletion of potentially self-reactive T cell receptor specificities in L3T4-, Lyt-2- T cells of lpr mice. J. Exp. Med. **168:** 2221–2229.

Matsumoto K., Yoshikai Y., Asano T., Himeno K., Iwasaki A., and Nomoto K. (1991) Defect in negative selection in lpr donor-derived T cells differentiating in non-lpr host thymus. J. Exp. Med. **173:** 127–136.

Mountz J.D., Smith T.M., and Toth K.S. (1990). Altered expression of self-reactive T cell receptor Vβ regions in autoimmune mice. J. Immunol. **144:** 2159–2166.

Murphy E.D., and Roths J.B. (1977). New inbred strains. Mouse Newslett. **58:** 51.

Ohteki T., Seki S., Abo T., and Kumagai K. (1990). Liver is a possible site for the proliferation of abnormal $CD3^+4^-8^-$ double-negative lymphocytes in autoimmune MRl-lpr/lpr mice. J. Exp Med. **172:** 7–12.

Santoro T.J., Portanova J.P., and Kotzin B.L. (1988). The contribution of L3T4+ T cells to lymphoproliferation and autoantibody production in MRL-lpr/lpr mice. J. Exp Med. **167:** 1713–1718.

Seki S., Abo T., Ohteki T., Sugiura K., and Kumagai K. (1991). Unusual αβ-T cells expanded in autoimmune lpr mice are probably a counterpart of normal T cells in the liver. J. Immunol. **147:** 1214–1221.

Simon M.M., Presten M., Nerg G., and Kuppers R.C. (1984). Quantitative studies on T cell function in MRL/Mp-lpr/lpr mice. Clin. Immunol. Immunopathol. **33:** 39–53.

Theofilopoulos A.N., and Dixon F.J. (1985). Murine models of systemic lupus erythematosus. Adv. Immunol. **37:** 269–390.

Woodland D., Happ M., Bill J., and Palmer E. (1990). Requirement for co-tolerogenic gene products in the clonal deletion of I-E reactive cells. Science **247:** 964–967.

Zhou T., Bleuthmann H., Eldridge J., Brockhaus M., Berry K., and Mountz J.D. (1991). Abnormal thymocyte development and production of autoreactive T cells in T cell receptor transgenic autoimmune mice. J. Immunol. **147:** 466–474.

